# The Relationship between C-Reactive Protein/Albumin Ratio and Disease Activity in Patients with Inflammatory Bowel Disease

**DOI:** 10.1155/2020/3467419

**Published:** 2020-06-22

**Authors:** Yi-Han Chen, Li Wang, Shu-Yi Feng, Wei-Min Cai, Xiao-Fu Chen, Zhi-Ming Huang

**Affiliations:** Department of Gastroenterology and Hepatology, The First Affiliated Hospital of Wenzhou Medical University, Wenzhou 325000, China

## Abstract

**Objectives:**

The aims of this study were to evaluate the C-reactive protein/albumin ratio (CRP/ALB), inflammatory markers, and parameters from the complete blood count (CBC) in patients with inflammatory bowel disease (IBD) and their associations with disease activity.

**Methods:**

A total of 876 IBD patients, composed of 275 patients with ulcerative colitis (UC) and 601 patients with Crohn's disease (CD), were included in this retrospective study, and the serum C-reactive protein (CRP), albumin (ALB), erythrocyte sedimentation rate (ESR), and CBC parameters were measured. To explore the disease activity, the Mayo score and Crohn disease activity index were used to assess UC and CD patients, respectively.

**Results:**

The CRP/ALB ratio, CRP, ESR, platelet to lymphocyte ratio (PLR), red blood cell distribution width (RDW), and neutrophil to lymphocyte ratio (NLR) levels in active IBD patients were significantly higher than those in inactive IBD patients, whereas ALB and lymphocyte to monocyte ratio (LMR) levels were significantly decreased (*P* < 0.001). The receiver operating characteristic analysis showed that the optimum cut-off values of the CRP/ALB ratio for active UC and CD were 0.18 and 0.43, with sensitivities of 67.8% and 75.8% and specificities of 86.7% and 92.0%, respectively. Multivariable logistic analysis revealed that after adjusting for these inflammatory markers (ESR, NLR, PLR, and LMR), the CRP/ALB ratio was a statistically significant parameter capable of differentiating the disease activity of UC and CD.

**Conclusions:**

This study indicated that the CRP/ALB ratio was closely related to the IBD disease activity. Compared with CBC parameters, the CRP/ALB ratio had a higher discriminative capacity for active IBD.

## 1. Introduction

Inflammatory bowel disease (IBD), a life-long disease resulting from the interaction of environmental and genetic elements, has been a global healthcare problem with a steadily increasing incidence [1]. IBD is mainly composed of two different bowel-relapsing disorders, including Crohn's disease (CD) and ulcerative colitis (UC). Early detection of the disease activity of IBD is of great significance for the treatment of this disease, which can effectively prevent complications and therefore improve prognosis as well as quality of life [2, 3]. Biomarkers of IBD can provide helpful information regarding the disease activity. Despite many efforts in the discovery of new biomarkers [4–7], endoscopy continues to be the gold standard for the diagnosis and evaluation of the disease activity in IBD patients. Thus, further studies are needed to find a cost-effective and noninvasive biomarker for clinical practice.

In current clinical practice, commonly used noninvasive biomarkers such as C-reactive protein (CRP) and erythrocyte sedimentation rate (ESR), are considered to be important for both early diagnosis and accurate monitoring of the disease activity in IBD patients [8]. As an acute phase protein, CRP measurements are widely available and relatively inexpensive to obtain; however, elevated serum CRP levels can also be affected by other extraintestinal inflammatory processes, and there is heterogeneity in the CRP production of individuals because of genetic differences [9]. ESR testing is less widely used than CRP testing due to the slower response to disease activity changes [10]. Fecal calprotectin has relatively better sensitivity and specificity and is the best clinical biological indicator for the assessment of the disease activity in IBD patients [11]; however, it is still not commonly used in clinical practice for multiple reasons including high cost, long time requirement, and the need to collect samples on demand. In the last few years, some systemic inflammatory markers obtained from the complete blood count (CBC), such as the neutrophil to lymphocyte ratio (NLR), platelet to lymphocyte ratio (PLR), and lymphocyte to monocyte ratio (LMR), have been reported as diagnostic and predictive indicators of IBD [5, 12, 13]. Moreover, a study showed that the red blood cell distribution width (RDW) can assess the disease activity of CD, but it does not perform well for UC [14]. Serum albumin (ALB) is generally recognized as an indicator of nutrition, and low ALB levels often indicate malnutrition. The rate of ALB synthesis is also directly affected by the severity of acute infection, given that ALB is a negative acute phase protein [15]. Furthermore, a recent study showed that ALB may have a diagnostic value in identifying children with potential CD [16].

The C-reactive protein/albumin ratio (CRP/ALB), determined by dividing the CRP level by the ALB level, was initially used as a novel predictor to identify critically ill patients in an acute medical ward [17]. In recent years, an increasing number of studies have suggested that an elevated CRP/ALB ratio predicts a worse prognosis in cancer patients [18]. Moreover, the CRP/ALB ratio has also been considered a useful biomarker for evaluating the disease activity in patients with Takayasu arteritis [19]. However, there have been few previous studies on the CRP/ALB ratio in IBD patients, and one study showed the association between the CRP/ALB ratio and the disease activity of CD [20].

Therefore, in this study, we evaluated the CRP/ALB ratio, inflammatory markers, and CBC parameters in patients with IBD and their associations with the disease activity.

## 2. Materials and Methods

### 2.1. Participants

From September 2011 to September 2019, a total of 876 IBD (275 UC, 601 CD) patients followed at the First Affiliated Hospital of Wenzhou Medical University were included in the current retrospective study. The study was approved by the hospital ethics committee. These patients were diagnosed with IBD based on standard clinical, laboratory, radiological, endoscopic, and histopathologic findings. Among 1511 IBD patients who were retrospectively analyzed, only 876 subjects met the criteria for inclusion in this study. Patients who met the following criteria were excluded: other autoimmune diseases, acute or chronic renal failure, heart diseases, cirrhosis, cancer, acute or chronic infections, and missing ALB or CRP data at admission. We extracted patient information, including age, sex, smoking history, body mass index (BMI), disease duration, endoscopic findings, localization of the disease, and detailed medication list (corticosteroids, 5-aminosalicylate (5-ASA), immunosuppressant, and biologics), from the medical records. The serum CRP level, ESR, ALB, and CBC parameters were also measured in all patients. The following ratios of inflammatory markers were calculated: CRP/ALB ratio, NLR, LMR, and PLR.

### 2.2. Disease Activity

The disease activity of UC and CD patients was evaluated by the Mayo score and Crohn disease activity index (CDAI), respectively. The Mayo score, a composite score incorporating 4 items: stool frequency, rectal bleeding, findings of flexible proctosigmoidoscopy, and physician's global assessment, was introduced more than 30 years ago and allows a simple and effective classification of UC patients [21, 22]. UC patients whose Mayo scores were >2 were divided into the active UC group. CD patients were categorized using the CDAI criteria, which includes weight, general health status, number of daily bloody stools, degree of abdominal pain, hematocrit, and complications [23]. CD patients with CDAI > 150 were divided into the active CD group.

### 2.3. Statistical Analysis

The Statistical Package for Social Sciences (SPSS) 22.0 for Windows was used for the statistical analyses. The normality of the distribution of continuous variables was determined by the Kolmogorov–Smirnov test. Continuous values were presented as the mean ± standard deviation or, in case of nonnormally distributed data, as the median and 25th–75th percentiles. The chi-square test was used to analyze categorical variables. Comparisons of normally distributed continuous variables were carried out using independent sample *t*-tests or paired *t*-tests. The Mann–Whitney *U* test for independent subgroups and the Wilcoxon test for dependent subgroups were used for the analysis of nonnormally distributed data. To determine the relationships of the CRP/ALB ratio and other laboratory parameters with the IBD activity, Spearman's correlation analysis was used. Multivariate logistic regression for the CRP/ALB ratio, ESR, NLR, PLR, and LMR was carried out to reveal which biomarkers were significantly associated with the disease activity in UC and CD patients. To differentiate between active and inactive patients with IBD, the optimal cut-off values of the CRP/ALB ratio, ALB, CRP, ESR, NLR, PLR, and LMR with maximum sensitivity and specificity were calculated via receiver operating characteristic (ROC) curve analysis. The *Z*-statistic was used to compare the areas under the ROC curves (AUCs). *P* < 0.05 was considered to be statistically significant.

## 3. Results

The total 876 IBD patients included in the study consisted of 275 UC patients and 601 CD patients. Among UC and CD patients, the median age was 48 and 27 years, respectively, with men accounting for 54.9% and 72.9%, respectively. Based on the criteria described above, 275 UC patients were divided into “UC remission” (98) and “UC active” (177), and 601 CD patients were divided into “CD remission” (299) and “CD active” (302). The demographic and clinical characteristics of subjects with UC and CD were shown in Table 1.

No significant differences in age or sex were observed between the active and inactive groups, in either the UC or CD patients (*P* > 0.05).  Table 2 shows that the CRP/ALB ratio, ESR, CRP, RDW, NLR, and PLR levels in the active UC group were significantly higher than those in the inactive UC group, whereas the ALB and LMR levels were significantly lower (*P* < 0.001). With respect to CD patients, BMI, CRP/albumin ratio, ESR, CRP, RDW, NLR, PLR, LMR, and ALB levels were also significantly different between patients with active disease and patients in remission (*P* < 0.001), as shown in Table 3.

As shown in Table 4, the results of the Spearman correlation analysis showed correlations between laboratory parameters and the IBD activity. ESR (*r* = 0.421, *P* < 0.001), CRP (*r* = 0.595, *P* < 0.001), and the CRP/ALB ratio (*r* = 0.637, *P* < 0.001) had significant correlations with the UC activity, while ALB (*r* = −0.692, *P* < 0.001) and LMR (*r* = −0.495, *P* < 0.001) were negatively associated with UC disease activity. In addition, the Spearman correlation analysis in CD patients also indicated significant positive correlations of the CD activity with ESR (*r* = 0.63, *P* < 0.001), CRP (*r* = 0.731, *P* < 0.001), and the CRP/ALB ratio (*r* = 0.763, *P* < 0.001) and negative correlations with ALB (*r* = −0.748, *P* < 0.001) and LMR (*r* = −0.454, *P* < 0.001).

The results of multivariate logistic regression analysis for exploring the associations of the CRP/ALB ratio, ESR, NLR, PLR, and LMR with active UC and CD were shown in Table 5. After adjusting for these inflammatory markers (ESR, NLR, PLR, and LMR), the odds ratios of the CRP/ALB ratio in UC and CD patients were 1.359 (95% confidence interval, 1.144-1.615) and 1.569 (95% confidence interval, 1.413-1.741), respectively. Therefore, the CRP/ALB ratio was an independent predictive factor of the disease activity of UC and CD.

**Figure 1:**
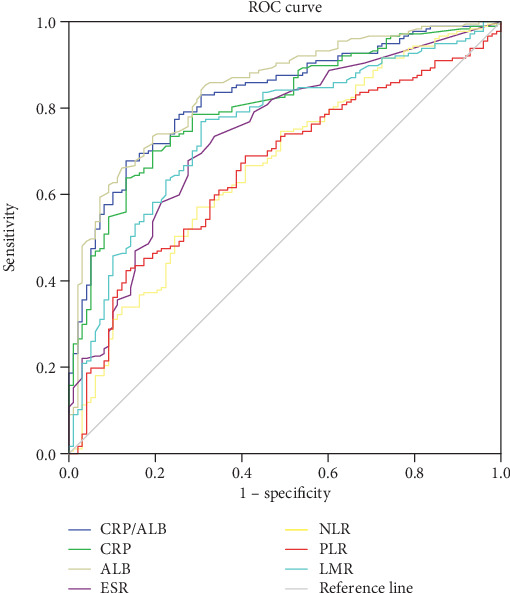
Receiver operating characteristic (ROC) curve of CRP/ALB vs. other inflammatory markers in predicting active UC.

**Figure 2:**
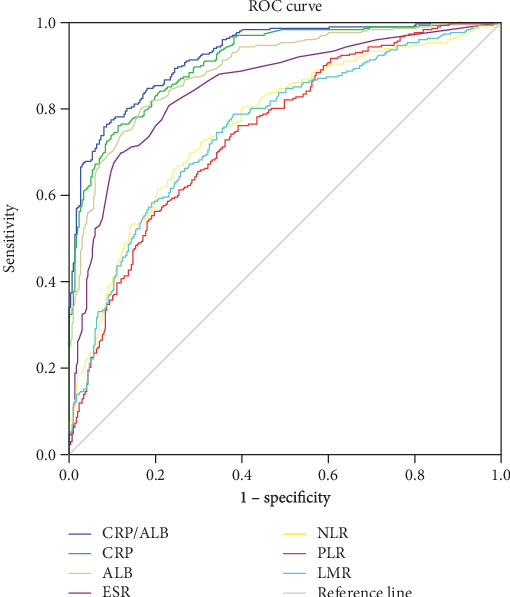
Receiver operating characteristic (ROC) curve of CRP/ALB vs. other inflammatory markers in predicting active CD.

The predictive values of ESR, CRP, CRP/ALB ratio, albumin, NLR, PLR, and LMR for differentiating active from inactive UC and CD were investigated by the receiver operating characteristic (ROC) curve analysis. The results of this analysis are shown in Tables 6 and 7. As shown in Figures 1 and 2, the discriminatory values of these indicators relative to the UC and CD activities were depicted. The results of ROC analysis showed that the AUC of ALB in UC patients was 0.845, which was higher than that of CRP (0.806) and the CRP/ALB ratio (0.827) (*P* > 0.05). However, we found that the AUC of the CRP/ALB ratio (0.925) was higher than that of CRP (0.91) and ALB (0.893) in CD patients (*P* > 0.05). It is worth noting that according to the *Z*-statistic, the AUCs of the NLR, PLR, LMR, and ESR were all lower than that of the CRP/ALB ratio in both UC and CD patients (*P* < 0.05).

## 4. Discussion

The aim of this retrospective study was to explore the use of the CRP/ALB ratio as a new noninvasive biomarker for evaluating the disease activity of IBD. In our study, significant increases in the CRP/ALB ratio, ESR, RDW, NLR, PLR, and CRP levels and significant decreases in LMR and ALB levels were observed in the active groups compared to the respective levels in the remitted IBD group. Furthermore, these parameters correlated strongly with UC and CD activities. In addition, we also found that among IBD patients, the BMI of active patients was lower than that of patients in remission (*P* < 0.05). This means that the nutritional status of patients in the active phase is worse, indicating that the nutritional status of patients is related to the disease activity. ROC analysis suggested that the AUC of ALB in UC patients was 0.845, which was higher than that of CRP (0.806) and the CRP/ALB ratio (0.827) (*P* > 0.05), but for patients with CD, the AUC of the CRP/ALB ratio (0.925) was higher than the AUCs of CRP (0.91) and ALB (0.893; *P* > 0.05). After adjusting for the other inflammatory markers (NLR, ESR, PLR, and LMR), the CRP/ALB ratio was an independent predictive factor for the disease activity of UC and CD.

The analysis of components of the CBC is a simple and inexpensive method to assess the disease activity of IBD. The association between CBC parameters and the disease activity in UC and CD patients has previously been studied [5, 6, 14]. It has previously been shown that in patients with active UC and CD, the RDW, the NLR, and the PLR were increased, and the LMR was decreased. These previous findings support the results of the present study. However, as previous studies reported that the NLR had no value for discriminating the disease activity, the role of the NLR in predicting the UC and CD disease activities remains controversial [24, 25]. Xu et al. [26] reported that a low LMR might be an effective biomarker for identifying the disease activity of UC, but this is not the case when evaluating the disease activity of CD. In our study, according to the results of multivariate logistic regression analysis, we found that LMR has a good value in differentiating the disease activity of UC, which is basically close to the findings of previous studies. Moreover, we found that the NLR was well correlated with the activity of CD, so there are still controversies about the NLR.

CRP and ALB, known as positive and negative acute phase reactants, respectively, are commonly used to assess inflammatory processes. Previous research also confirms that the inflammatory response can influence ALB synthesis [27]. The CRP/ALB ratio is considered a novel inflammation-based score that provides more useful information about inflammatory status than CRP or albumin alone in septic patients [28]. Qin et al. [20] found that the CRP/ALB ratio and ALB levels had a predictive value in determining the CD disease activity and were more significant in men. The retrospective study of 100 patients with active or inactive CD showed that the optimal cut-off point of the CRP/ALB ratio for active CD was 0.69, with a sensitivity and specificity of 59.7% and 81.6%, respectively. In our study, we found that the optimal cut-off point of the CRP/ALB ratio for active CD was 0.43, with a sensitivity of 75.8% and a specificity of 92%, and the AUC of the CRP/ALB ratio was 0.925, which was higher than that of ALB (0.893; *P* > 0.05). The sensitivity and specificity of the CRP/ALB ratio in this study were higher than those in a previous study, which we consider to be mainly because the CD patients in the remission period we included had a milder status. In the present study, the CRP/ALB ratio showed a stronger correlation with the disease activity in IBD patients than the components of the CBC. According to the results of the *Z* statistic, the AUCs of the NLR, PLR, LMR, and ESR were all lower than that of the CRP/ALB ratio in both UC and CD patients (*P* < 0.05). In addition, the results of the multivariate analysis evaluating statistically significant parameters suggested that the CRP/ALB ratio was an important parameter capable of determining the disease activity of UC and CD.

Our study had some limitations. First, the patients we included were all followed at our hospital in southeastern China. Therefore, the results may not represent the general population in China. Second, our study was not designed to elucidate the mechanism that led to the observed increases of the CRP/ALB ratio in patients with active UC and CD. Third, this study did not evaluate other factors, such as the use of immunosuppressive agents and corticosteroids, which may affect the level of inflammatory markers. Finally, our study was designed as a retrospective single-center study, which prevents firm conclusions from being drawn. Prospective studies are needed to provide more useful information on this subject.

## 5. Conclusions

The findings from this study demonstrate that in subjects with IBD, the CRP/ALB ratio was strongly related to the disease activity. Compared with parameters from the CBC, the CRP/ALB ratio had a higher discriminative capacity for active IBD. After adjusting for these inflammatory markers (ESR, NLR, PLR, and LMR), the CRP/ALB ratio was a helpful biomarker to differentiate the disease activity of UC and CD.

## Figures and Tables

**Table 1:** Characteristics of the patients in the UC and CD cohorts.

	UC (*n* = 275)	CD (*n* = 601)
Age (years)	48 (36-61)	27 (22-33)
Sex (F/M)	124/151	163/438
Smoking (yes/no)	47/228	90/511
BMI (kg/m^2^)	20.06 (18.44-21.28)	19.33 (17.44-20.9)
Duration of disease (months)	15.83 (6-48)	22.07 (7.3-43.88)
Remission	98 (35.6%)	299 (49.8%)
Active disease	177 (64.4%)	302 (50.2%)
Localization of disease		
Proctitis	49 (17.8%)	—
Left-side colitis	122 (44.4%)	—
Extensive colitis	104 (37.8%)	—
Terminal ileitis	—	139 (23.1%)
Colitis	—	117 (19.5%)
Ileocolitis	—	345 (57.4%)
Medication history		
Steroids	102 (37.1%)	267 (44.4%)
5-ASA	234 (85.1%)	468 (77.9%)
Immunosuppressant	31 (11.3%)	283 (47.1%)
Biologics	22 (8.0%)	337 (56.1%)

Values are expressed as *n* (%) or median (IQR). UC: ulcerative colitis; CD: Crohn's disease; BMI: body mass index.

**Table 2:** Differences between active disease and remission in UC patients.

Parameters	UC active (*n* = 177)	UC remission (*n* = 98)	*P* value
Age (years)	49 (37-61)	46.5 (33.25-61)	0.323
Sex (F/M)	76/101	48/50	0.335
Smoking (yes/no)	30/147	17/81	0.933
BMI (kg/m^2^)	19.27 (18.27-20.81)	20.64 (18.83-21.89)	<0.001
ESR (mm/h)	22 (10.5-37.5)	6 (2-18)	<0.001
CRP (mg/L)	12.7 (3.65-32.55)	2.94 (1.26-4.09)	<0.001
ALB (g/L)	34.2 ± 5.9	41.7 ± 4.5	<0.001
CRP/ALB	0.36 (0.1-0.97)	0.06 (0.03-0.1)	<0.001
Neutrophil (10^9^/L)	4.6 (3.42-7.03)	3.55 (2.7-4.93)	<0.001
Monocyte (10^9^/L)	0.7 (0.46-0.92)	0.48 (0.38-0.63)	<0.001
Lymphocyte (10^9^/L)	1.76 (1.33-2.23)	1.91 (1.5-2.49)	0.063
Hgb (g/L)	119 (103.5-132)	131 (120.75-139)	<0.001
MCV (fl)	88.9 (82.85-92.75)	89 (86.57-93.1)	0.035
RDW (%)	14 (13.45-15.05)	13.3 (12.7-14.3)	<0.001
Platelet (10^9^/L)	281 (210-374)	236 (199.25-284)	<0.001
MPV (fl)	9.9 (9.3-11)	10.3 (9.6-11.23)	0.074
NLR	2.82 (1.77-4.21)	1.84 (1.32-2.81)	<0.001
PLR	163.38 (121.28-222.53)	122.74 (92.31-168.64)	<0.001
LMR	2.67 (1.8-3.5)	4.16 (3.17-5.14)	<0.001

Values are expressed as the mean ± SD or median (IQR). UC: ulcerative colitis; BMI: body mass index; ESR: erythrocyte sedimentation rate; CRP: C-reactive protein; ALB: albumin; MCV: mean corpuscular volume; RDW: red cell distribution width; MPV: mean platelet volume; NLR: neutrophil to lymphocyte ratio; PLR: platelet to lymphocyte ratio; LMR: lymphocyte to monocyte ratio.

**Table 3:** Differences between active disease and remission in CD patients.

Parameters	CD active (*n* = 302)	CD remission (*n* = 299)	*P* value
Age (years)	26 (22-35)	27 (22-31)	0.910
Sex (F/M)	81/221	82/217	0.868
Smoking (yes/no)	51/251	39/260	0.187
BMI (kg/m^2^)	18.16 (16.55-20.15)	19.56 (18.25-21.6)	<0.001
ESR (mm/h)	30.5 (17-47)	7 (2-14)	<0.001
CRP (mg/L)	30 (14.9-59.8)	3.07 (1.89-9.09)	<0.001
ALB (g/L)	33.3 ± 5.0	41.7 ± 4.7	<0.001
CRP/ALB	0.88 (0.43-1.86)	0.07 (0.04-0.22)	<0.001
Neutrophil (10^9^/L)	5.01 (3.48-7.07)	3.46 (2.61-4.23)	<0.001
Monocyte (10^9^/L)	0.69 (0.47-0.9)	0.51 (0.39-0.63)	<0.001
Lymphocyte (10^9^/L)	1.25 (0.9-1.67)	1.57 (1.14-1.95)	<0.001
Hgb (g/L)	115 (101-127)	135 (123-149)	<0.001
MCV (fl)	83.25 (78.43-87.6)	88.4 (84.8-92.1)	<0.001
RDW (%)	14.8 (13.3-16.53)	13.3 (12.7-14.7)	<0.001
Platelet (10^9^/L)	327.5 (253.75-410.5)	246 (206-294)	<0.001
MPV (fl)	9.6 (8.9-10.4)	10.6 (10-11.4)	<0.001
NLR	4.08 (2.78-6.13)	2.17 (1.52-3.31)	<0.001
PLR	264.84 (192.08-367.64)	165.61 (118.72-230)	<0.001
LMR	1.89 (1.36-2.57)	3.05 (2.27-4.25)	<0.001

Values are expressed as the mean ± SD or median (IQR). CD: Crohn's disease; BMI: body mass index; ESR: erythrocyte sedimentation rate; CRP: C-reactive protein; ALB: albumin; MCV: mean corpuscular volume; RDW: red cell distribution width; MPV: mean platelet volume; NLR: neutrophil to lymphocyte ratio; PLR: platelet to lymphocyte ratio; LMR: lymphocyte to monocyte ratio.

**Table 4:** Spearman correlation coefficients between laboratory parameters and the disease activity of UC and CD.

Parameters	Mayo UC score		CDAI	
	*r* value	*P* value	*r* value	*P* value
ESR	0.421	<0.001	0.630	<0.001
CRP	0.595	<0.001	0.731	<0.001
ALB	-0.692	<0.001	-0.748	<0.001
CRP/ALB	0.637	<0.001	0.763	<0.001
Neutrophil	0.334	<0.001	0.370	<0.001
Monocyte	0.354	<0.001	0.270	<0.001
Lymphocyte	-0.203	0.001	-0.241	<0.001
Hgb	-0.402	<0.001	-0.598	<0.001
MCV	-0.212	<0.001	-0.389	<0.001
RDW	0.341	<0.001	0.370	<0.001
Platelet	0.243	<0.001	0.369	<0.001
MPV	-0.144	0.017	-0.449	<0.001
NLR	0.393	<0.001	0.451	<0.001
PLR	0.350	<0.001	0.442	<0.001
LMR	-0.495	<0.001	-0.454	<0.001

UC: ulcerative colitis; CD: Crohn's disease; CDAI: Crohn's disease activity index; ESR: erythrocyte sedimentation rate; CRP: C-reactive protein; ALB: albumin; MCV: mean corpuscular volume; RDW: red cell distribution width; MPV: mean platelet volume; NLR: neutrophil to lymphocyte ratio; PLR: platelet to lymphocyte ratio; LMR: lymphocyte to monocyte ratio.

**Table 5:** Results of multivariate logistic regression analysis in patients with UC and CD.

	Variables	*B*	*P*	Odds ratio	95% CI
UC	CRP/ALB	0.307	<0.001	1.359	1.144-1.615
	ESR	0.021	0.087	1.021	0.997-1.045
	NLR	-0.085	0.304	0.919	0.782-1.080
	PLR	0.004	0.156	1.004	0.999-1.009
	LMR	-0.247	0.002	0.781	0.666-0.915

CD	CRP/ALB	0.450	<0.001	1.569	1.413-1.741
	ESR	0.029	0.001	1.029	1.011-1.047
	NLR	0.160	0.026	1.174	1.019-1.351
	PLR	0.001	0.235	1.001	0.999-1.004
	LMR	-0.031	0.657	0.969	0.844-1.113

UC: ulcerative colitis; CD: Crohn's disease; ESR: erythrocyte sedimentation rate; NLR: neutrophil to lymphocyte ratio; PLR: platelet to lymphocyte ratio; LMR: lymphocyte to monocyte ratio; CI: confidence interval.

**Table 6:** Accuracy of CRP/ALB and other inflammatory markers in differentiating active from inactive UC.

Parameters	AUCs	SE	95% CI	Cut-offs	Sensitivity (%)	Specificity (%)
CRP/ALB	0.827	0.025	0.777-0.870	0.18	67.8	86.7
CRP	0.806	0.026	0.754-0.851	5.38	80.6	70.1
ALB	0.845	0.024	0.797-0.885	38.20	73.5	80.6
ESR	0.739	0.031	0.683-0.790	15.00	67.8	72.5
NLR	0.665	0.034	0.606-0.721	2.40	57.1	70.4
PLR	0.660	0.034	0.601-0.716	187.68	42.4	86.7
LMR	0.752	0.030	0.697-0.802	3.56	76.8	69.4

UC: ulcerative colitis; AUC: area under the curve; SE: standard error; CI: confidence interval; CRP: C-reactive protein; ALB: albumin; MCV: mean corpuscular volume; ESR: erythrocyte sedimentation rate; NLR: neutrophil to lymphocyte ratio; PLR: platelet to lymphocyte ratio; LMR: lymphocyte to monocyte ratio.

**Table 7:** Accuracy of CRP/ALB and other inflammatory markers in differentiating active from inactive CD.

Parameters	AUCs	SE	95% CI	Cut-offs	Sensitivity (%)	Specificity (%)
CRP/ALB	0.925	0.010	0.901-0.945	0.43	75.8	92.0
CRP	0.910	0.011	0.884-0.932	14.70	76.2	88.6
ALB	0.893	0.013	0.866-0.917	36.80	80.1	83.3
ESR	0.851	0.016	0.820-0.878	20.90	69.9	88.0
NLR	0.764	0.019	0.728-0.798	3.32	65.9	75.9
PLR	0.747	0.020	0.710-0.781	191.22	76.2	60.9
LMR	0.755	0.020	0.719-0.789	2.67	78.8	61.9

CD: Crohn's disease; AUC: area under the curve; SE: standard error; CI: confidence interval; CRP: C-reactive protein; ALB: albumin; MCV: mean corpuscular volume; ESR: erythrocyte sedimentation rate; NLR: neutrophil to lymphocyte ratio; PLR: platelet to lymphocyte ratio; LMR: lymphocyte to monocyte ratio.

## Data Availability

The data used to support the findings of this study are available from the corresponding author upon request.
